# Molecular Dynamics Insight into the Lipid II Recognition by Type A Lantibiotics: Nisin, Epidermin, and Gallidermin

**DOI:** 10.3390/mi12101169

**Published:** 2021-09-28

**Authors:** Irina Panina, Amir Taldaev, Roman Efremov, Anton Chugunov

**Affiliations:** 1 Shemyakin-Ovchinnikov Institute of Bioorganic Chemistry, Russian Academy of Sciences, 16/10 Miklukho-Maklaya St., 117997 Moscow, Russia; irinaspanina@gmail.com (I.P.); r-efremov@yandex.ru (R.E.); 2 Moscow Institute of Electronics and Mathematics, National Research University Higher School of Economics, 101000 Moscow, Russia; 3 Institute of Urology and Reproductive Health, Sechenov First Moscow State Medical University (Sechenov University), 8-2 Trubetskaya St., 119991 Moscow, Russia; t-amir@bk.ru; 4 V.N. Orekhovich Institute of Biomedical Chemistry, 10-8 Pogodinskaya St., 119121 Moscow, Russia

**Keywords:** lantibiotics, nisin, epidermin, gallidermin, pyrophosphate pharmacophore, molecular dynamics, molecular recognition

## Abstract

Lanthionine-containing peptides (lantibiotics) have been considered as pharmaceutical candidates for decades, although their clinical application has been restricted. Most lantibiotics kill bacteria *via* targeting and segregating of the cell wall precursor—membrane-inserted lipid II molecule—in some cases accompanied by pores formation. Nisin-like lantibiotics specifically bind to pyrophosphate (PPi) moiety of lipid II with their structurally similar N-terminal thioether rings A and B. Although possessing higher pore-forming capability, nisin, in some cases, is 10-fold less efficient *in vivo* as compared to related epidermin and gallidermin peptides, differing just in a few amino acid residues within their target-binding regions. Here, using molecular dynamics simulations, we investigated atomistic details of intermolecular interactions between the truncated analogues of these peptides (residues 1–12) and lipid II mimic (dimethyl pyrophosphate, DMPPi). The peptides adopt similar conformation upon DMPPi binding with backbone amide protons orienting into a single center capturing PPi moiety *via* simultaneous formation of up to seven hydrogen bonds. Epidermin and gallidermin adopt the complex-forming conformation twice as frequent as nisin does, enhancing the binding by the lysine 4 side chain. Introduction of the similar residue to nisin *in silico* improves the binding, providing ideas for further design of prototypic antibiotics.

## 1. Introduction

Given the rising growth of antibiotic resistance, identifying and developing novel classes of antibacterial drugs with new mechanisms of action are urgently required [1,2,3]. Bacteria produce a variety of bioactive molecules to kill other strains, most of which differ from the marketed antibiotics. Lantibiotics are a class of lanthionine-containing antimicrobial peptides (AMPs), which are considered potent antibacterial drug candidates due to the conserved chemical structure of their target. Most of them are produced by and are effective mainly against Gram-positive bacteria [4,5]. Lantibiotics undergo substantial post-translational modifications that are important for antimicrobial activity. As a result, these peptides have complex thioether rings introduced by the modified amino acids lanthionine (Lan) and/or methyllanthionine (MeLan) as well as a number of non-canonical residues, such as dehydroalanine (Dha) and dehydrobutyrine (Dhb) (Figure S1) [6,7].

The current classification divides lantibiotics into two major groups based on their structure, mechanism of action, and post-translational modification: (1) elongated and positively charged type A peptides (exemplified by nisin); (2) globular and negatively charged/neutral type B peptides (such as mersacidin) [8,9]. Additionally, some lantibiotics consist of two different peptides, each being AMP by themselves, yet synergistically active together. These are often identified as type C (for example, lichenicidin) [10,11,12,13].

**Figure 1:**
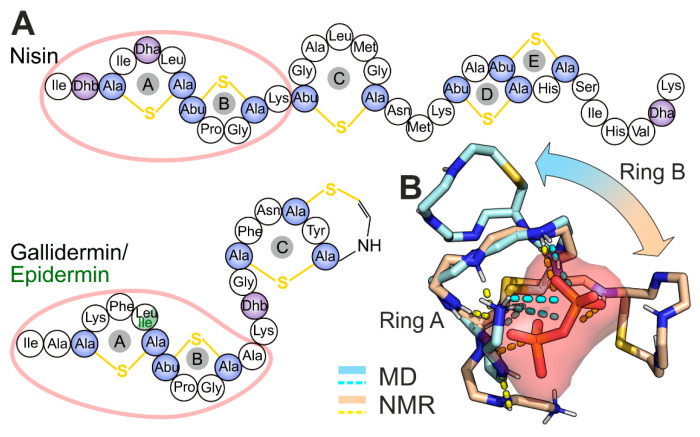
Structures of nisin, epidermin, gallidermin, and nisin/lipid II complexes in water and DMSO. (**A**) Schematic chemical structure of nisin, epidermin, and gallidermin. Modified amino acids are colored: unsaturated Dha and Dhb amino acids in *purple*; Lan and MeLan in *blue* (see also Figure S1). Note a single amino acid substitution, which differs gallidermin and epidermin. (**B**) Spatial structure of nisin/lipid II complex: obtained by NMR in DMSO [21] (*wheat*) and predicted by molecular dynamics simulations in water solution (*pale blue*) [27]. The major discrepancy (ring B relative position) is highlighted with an *arrow*. The peptide backbone is shown with *sticks*, wherein carbon—*pale blue/wheat*, nitrogen—*blue*, hydrogen—*white*, and sulfur—*yellow* side chains and oxygen atoms are hidden for clarity. The pyrophosphate moiety is represented by *red* and *orange sticks* and *surface*. The PPi location and conformation are similar and represented by an averaged MD-derived structure.

Nisin [14] (Figure 1A), the most studied type A lantibiotic, is characterized by a dual mode of action: (1) it targets bacterial peptidoglycan precursor lipid II (Figure 1B), withdrawing it from the cell wall biosynthesis and, thus, inhibiting growth of the bacteria [15]. (2) Furthermore, binding to lipid II promotes pore formation in the cell membrane, leading to the lysis of the bacteria [16,17,18]. Nisin:lipid II stoichiometry in the pore complex is presumably 8:4 [19].

NMR studies revealed that the N-terminus of nisin is responsible for lipid II recognition and binding, while the C-terminal part is involved in the pore formation [20]. The only resolved complex structure of nisin and shortened lipid II analogue in DMSO (pdb ID: 1WCO) disclosed the binding motif: backbone amides of A and B rings wrap pyrophosphate moiety (PPi) of lipid II through five hydrogen bonds in a cage-like configuration [21]. The binding of nisin to the conserved lipid II’s PPi group, which is unlikely to be changed, makes this peptide a promising prototype of a new therapeutic agent. More recent solid-state NMR studies of nisin/lipid II interaction in model liposomes and bacterial membranes demonstrate that a complex structure within the lipid environment considerably contradicts that found in DMSO [22], yet does not deliver the alternative structure of the complex.

Other type A lantibiotics—nisin’s structural analogues epidermin [23] and gallidermin [24]—have a similar N-terminal A/B-ring lipid II-binding motif (Figure 1A) and have displayed a similar mechanism of bactericidal action [17,25]. Despite being substantially shorter (22 *vs.* 34 amino acids as compared to nisin), both epidermin and gallidermin were shown to possess pore-forming activity, depending on bacterial membrane thickness [26]. An *in vivo* assay demonstrated that nisin and gallidermin have comparable MICs against several strains (*Micrococcus flavus* DSM 1790 and *Staphylococcus simulans* 22), although gallidermin was less potent in an inducing potassium leakage (model experiment to assess the pore-forming activity). Moreover, the superior activity of epidermin (MIC value of 0.002 μM) and gallidermin (0.005 μM) over nisin (0.048 μM) was revealed against *Lactococcus lactis* subsp. *cremoris* HP, for which only nisin was shown to form pores in the membrane [26]. This result shows that pore formation is not a necessary component of the bactericidal action: lipid II withdrawal is, in many cases, enough, and nisin may not be the champion in this challenge.

The importance of the lipid II capture alone (without pore formation) has also been confirmed for the truncated (residues 1–12) nisin analogue, retaining bacteriostatic (but not antimicrobial) activity [28,29], in spite of the absence of the pore-forming module. Moreover, nisin_1–12_ analogue comprised dicarba instead of lanthionine bridges and its Dhb2Ala/Dha5Ala mutant also binds lipid II, as demonstrated by inhibition of the native nisin pore-forming activity [29]. Thus, lantibiotics containing similar N-terminal AB-ring systems offer a solid framework for the design of novel peptide antibiotics.

Molecular dynamics (MD) simulation is a useful technique to obtain the major conformations of proteins and their targets, together with the energetical characteristics of intermolecular interactions. MD modeling accompanied by free energy calculations can assist prediction of the binding affinity, promoting the development of new protein or ligand variants with advanced binding properties [30,31]. MD simulations and molecular docking were used to examine interactions between lipid II and lantibiotics, such as nisin (for more details see [32]), mutacin1140 [30,33], and lacticin 3147 [33]. Our previous MD study determined that conformational space of nisin’s AB-rings crucially depends on the molecular environment and is not essentially affected by the absence of the C-terminal CDE-rings [27]. Our simulations determined the structure of the nisin/lipid II complex in DMSO similar to the NMR structure [21] and predicted the alternative complex in water solution (Figure 1B), in which only ring A binds PPi, while ring B stabilizes this conformation *via* two inter-ring h-bonds. This configuration remained stable in a long-term MD run in the model bacterial membrane [27], and was confirmed in the NMR study on nisin_1–12_ in solution [34].

In this study, we aimed to:

1. Discover the general lipid II recognition pharmacophore in the three related peptides: nisin_1–12_, epidermin_1–12_, and gallidermin_1–12_. This was done by the MD simulations in water in presence and absence of the dimethyl pyrophosphate (DMPPi), which mimics the lipid II binding site, revealing the mutual adaptation of the peptides and their target.
2. Reveal sequence features of gallidermin and epidermin, which offer superior biological activity (in some cases) as compared to nisin.
3. Verify if this feature upon transfer to the nisin backbone increases its activity (*in silico*).

We propose a general pharmacophore for lipid II recognition, which may be shared by the most nisin-like AMPs. This knowledge may be used for discovery and design of novel polycyclic AMPs with improved antimicrobial properties.

## 2. Materials and Methods

### Molecular Dynamics Simulations

MD simulations with imposed periodic boundary conditions were performed using the GROMACS package version 2020.4 [35] and modified GROMOS 43a2x parameters set. Each simulation was performed using a unified MD protocol. The integration time step was of 2 fs. The van-der-Waals interactions were calculated using a 12 Å spherical cut-off function; electrostatic interactions were computed using the particle mesh Ewald algorithm [36] with a real space cut-off 12 Å. Temperature of 315 K and isotropic pressure of 1 bar were maintained using a V-rescale thermostat [37] and the Berendsen coupling method [38] (peptides and the solvent were coupled separately). The LINCS algorithm was employed to constrain all bonds to their correct lengths [39].

In this study, we performed the set of five independent MD simulations for the following solvated molecules: nisin_1–12_, epidermin_1–12_, and gallidermin_1–12_ without the ligand, and (in order to examine mutual adaptation of the peptides and their target) in presence of one or three DMPPi ions (Table 1). Initial coordinates of nisin_1–12_ were taken from MD-equilibrated states of the full-length molecule that has been published previously (NF1 state) [27]. Epidermin_1–12_ and gallidermin_1–12_ starting structures were prepared manually applying the standard mutagenesis utility of the PyMOL program version 2.5.0 (www.pymol.org, accessed on 31 August 2021) to the nisin_1–12_ starting conformation. The peptide was centered in the cubic box (typical size of 60 × 60 × 60 Å^3^) and solvated with SPC water molecules [40] and the required number Na^+^ or Cl^−^ ions to maintain electroneutrality. DMPPi molecules were randomly placed in a box with a minimum distance to the peptide of 10 Å.

The simulated systems were first equilibrated by energy minimization (1000 conjugate gradient steps) followed by gradual heating from 5 to 315 K during 200-ps MD run with fixed heavy atoms of peptide/DMPPi. After equilibration, 500-ns MD production runs were carried out for each system and repeated 4 or 5 times by randomly assigning initial velocities. Total duration of the MD simulations in this study was ≈27 μs.

The analysis of MD trajectories was performed using standard GROMACS utilities and custom python scripts. The root-mean-square fluctuation (RMSF), inter- and intramolecular H-bonds profiles were calculated using GROMACS built-in tools. Cluster analysis was performed for all peptides, both in presence and in absence of DMPPi, using the merged data from 5 MD replicas and the gmx *cluster* module, where backbone atoms of residues 3–11 were superimposed. The GROMOS clustering method with cut-off of 2.2 Å was applied, and the largest clusters (>1%) were extracted.

## 3. Results

### 3.1. Nisin, Epidermin, and Gallidermin Reveal Similar Binding Motif

To investigate the peptides’ conformational ensembles, as well as the structural changes upon target binding, we performed a series of 500-ns MD simulations of nisin_1–12_, epidermin_1–12_, and gallidermin_1–12_ in an aqueous solution in the presence and absence of DMPPi, which mimics the binding site of lipid II (for simulations summary, see Table 1). Simulation of realistic models of peptides/lipid II complexes at the surface of the bacterial membrane is highly complicated because of a sampling bias. The parent for the lantibiotics water environment seems to be suitable for investigation of the basic principles of highly selective pyrophosphate recognition by registering conformational changes.

In the absence of the ligand, all peptides exhibit similar conformational ensembles: cluster analysis over the residue 3–11 backbones revealed six conformations for nisin_1–12_ (N1–6, Figure S2A) and epidermin_1–12_ (E1–6, Figure S2B), and seven for gallidermin_1–12_ (G1–7, Figure S2C). The conformational ensemble obtained for nisin_1–12_ is in agreement with the NMR data on this fragment in DMSO [34] and our previous calculations for nisin_1–11_ [27]. Moreover, among each peptide’s ensemble, one can find the lipid II-bound state observed in DMSO (pdb ID: 1WCO) [21], although with different populations: N3 (7.4%), E6 (3.1%), and G7 (3.0%). Conformational diversity is accounted for relative position of the rings A and B, characterized by backbone rotation between residues 7 and 8. The identical among the three peptides ring B is conformationally invariant (backbone (bb) RMSF is less than 0.7 Å). Ring A is more flexible, although it is more rigid in the nisin case (RMSF_bb_ 0.6–1 Å), as compared to epidermin and gallidermin (RMSF_bb_ 1.1–1.4 Å; see Figure S3).

**Figure 2:**
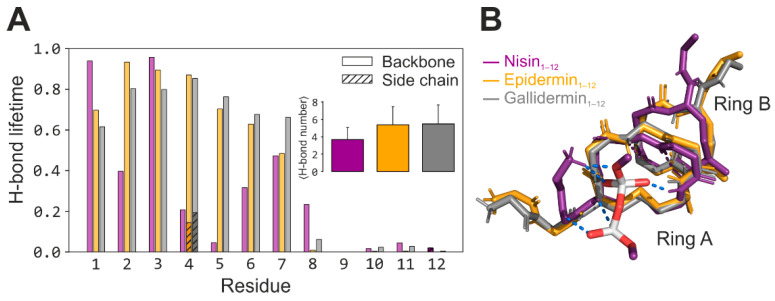
Binding modes of nisin, epidermin, and gallidermin to DMPPi revealed in MD simulations. (**A**) Intermolecular H-bonds lifetimes (as a fraction of MD time) per each residue. Note the contribution from the Lys 4 side chain for epidermin and gallidermin (*hatched bars*). *Inset:* average H-bonds number (±s.d.) over the whole peptides_1–12_ in MD. (**B**) Representative structures of the most populated clusters. The structures are superimposed over the backbone of the residues 3–11. For clarity, only nisin-bound DMPPi is shown with *sticks*, wherein oxygen—*red*, phosphorus—*white*, carbon—*purple*; peptides side chains and oxygen atoms are hidden. H-bonds are depicted with *blue dotted lines*.

Upon addition of the DMPPi ion(s), the peptide/pyrophosphate complex formed spontaneously and immediately, regardless of the number of ions (one or three). The DMPPi binding site is localized within ring A and N-terminus. Ring B is infrequently involved in PPi capturing, unlike the NMR structure in DMSO, where ring B is the principal binder (Figures S4–S6). The excess of the ligand (3 DMPPi per system) results in rare and unstable binding of the second DMPPi ion to ring B (Figures S7–S9). Substrate binding restricted the peptides’ flexibility: a total of five, three, and three major states were observed for DMPPi-bound nisin_1–12_ (NB1–5), epidermin_1–12_ (EB1–3), and gallidermin_1–12_ (NG1–3), respectively (*vs.* six, six, and seven states for the unbound peptides; compare Figures S2 and S10). Notably, epidermin and gallidermin exhibit pronounced ring A stabilization upon target binding, which is reflected in predominant occupancy of the first cluster (89.2% and 73%, accordingly) and marked reduction of ring A RMSF values (RMSF_bb_ 0.7–1 Å, Figure S3). All peptides shared a similar binding motif: backbone amide protons of mainly residues 1–7 orienting into a single center to capture the PPi moiety *via* the simultaneous formation of intermolecular H-bonds (2–5 for nisin and 3–8 for epidermin and gallidermin; Figure 2A *inset*). This interaction type determines the interaction, in addition to a salt bridge between a negatively charged pyrophosphate and positively charged peptide N-terminus.

The most populated complex-forming conformations of these peptides (NB1, EB1, and GB1) exhibit highly similar folds (Figure 2B). In this state, rings A and B are pulled together by the inter-ring H-bond between residues 5 and 8. Nisin_1–12_ forms an additional H-bond Dha5–Pro9 (Figure S11). According to this and our previous study [27], this state fits most for DMPPi binding, in contrast to the well-known NMR structure of the complex determined in the DMSO medium [21]. In this state, the number of intermolecular H-bonds to the captured DMPPi ion over the MD is 4 ± 1.1 (nisin_1–12_), 5.5 ± 2 (epidermin_1–12_) and 5.8 ± 2 (gallidermin_1–12_), exhibiting a solid superiority of the two latter peptides.

### 3.2. The Source of Epidermin and Gallidermin Advantage over Nisin

Despite the high structural similarity of the three peptides and their DMPPi complexes, there are at least two structural aspects that determine the superiority of epidermin and gallidermin binding over nisin. This advantage arises from an increased plasticity of the ring A in epidermin and gallidermin, indicated by RMSF values (Figure S3). Nisin’s sp^2^-hybridized C_α_-atom in Dha-5 renders its ring A enormously rigid, turning the Dha-5’s NH-group away from the PPi binding site. On the contrary, all five residues of ring A in epidermin and gallidermin form H-bonds with the DMPPi ion (Figure 2A and Figures S4–S9).

**Figure 3:**
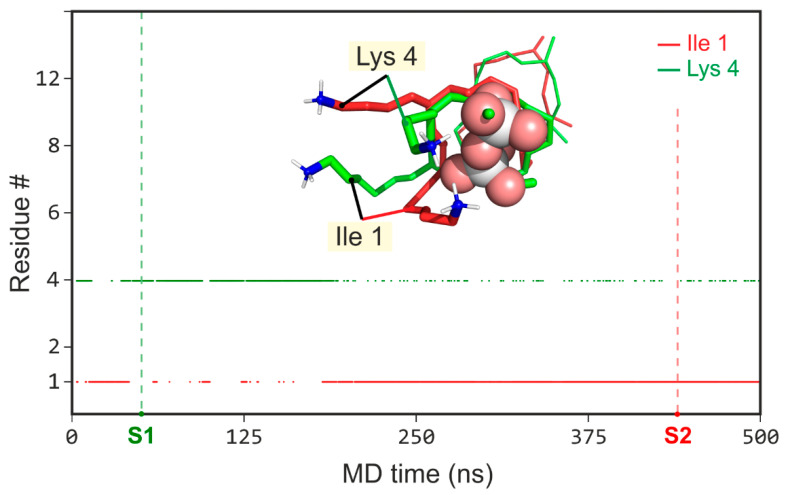
The alternate charge in the gallidermin’s DMPPi binding site. The main graph is a time-dependent H-bonding pattern between DMPPi ion and the charged groups of gallidermin_1–12_: Ile1 (*red*) and Lys4 (*green*). *Inset*: zoomed-in spatial structure of two binding regimens in the major bound state of galligermin_1–12_: S1 (*green*)—ε-NH^3+^-group of Lys4 binds to DMPPi; S2 (*red*)—*N*-terminus (NH^3+^-group of Ile1) binds to DMPPi. DMPPi is shown with *spheres* and *sticks*, wherein oxygen—*red*, phosphorus—*white*, carbon—*green*. For epidermin_1–12_, the same applies.

Moreover, the nisin_1–12_’s binding site has a single (N-terminal) positively charged group, while epidermin_1–12_ and gallidermin_1–12_ possess an alternative NH^3+^-group of Lys4 side chain. In the course of MD trajectories, charged groups of Ile1 and Lys4 interchangeably interact with DMPPi, wrapping it from the opposite to the ring’s planeside (for gallidermin example, see Figure 3). The established correlation coefficient (R) showing either NH^3+^ group of Ile1 or Lys4 or both bind to DMPPi (Figure 3) revealed strong anti-correlation: R = −0.96/−0.92 for gallidermin_1–12_ GB1 and epidermin_1–12_ EB1 states, respectively.

### 3.3. Single Mutation May Improve Nisin Binding Ability

Our comparative *in silico* analysis of nisin_1–12_, epidermin_1–12_, and gallidermin_1–12_ reveals slight, yet important differences in the way these peptides bind to DMPPi. The results are well consistent with, and may interpret the experimental data on different *in vivo* activities [26]. Given the assumption that enhanced binding of epidermin_1–12_ and gallidermin_1–12_ resulted from the additional Lys-4’s ε-NH^3+^ group and the absence of a flat part of the ring A, we introduced a point mutation (Dha5Lys) into nisin_1–12_, which combines both advantages. In order to examine the impact of the point mutation, we performed the same set of MD simulations of the mutant in the presence of DMPPi (Table 1).

**Figure 4:**
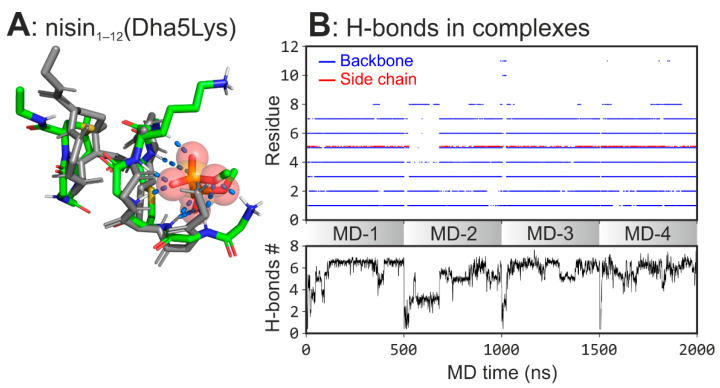
Spontaneous formation of nisin_1–12_(Dha5Lys)/DMPPi complexes in MD. (**A**) The representative structure of the complex. (**B**) *Upper panel:* hydrogen bonds map between nisin_1–12_(Dha5Lys) and DMPPi ions. Each dot indicates the H-bond between DMPPi and a particular residue backbone amide group (*blue*) or side chain (*red*) (*along the vertical axis*) at a given MD time (*along the horizontal axis*). *Lower panel:* the time-averaged (0.1 ns window) number of H-bonds between nisin_1–12_(Dha5Lys) and DMPPi. Both panels are stacked plots for four independent MD replicas (MD-1–4).

Introduction of the point mutation shifts the conformational equilibrium towards the higher population of the complex-forming state (64.8% *vs.* 51.8% for NB1 of nisin_1–12_). The hydrogen bonds analysis showed that Dha5Lys mutation increases the ring A flexibility and improves interaction with the ligand. Reduced stiffness of the binding site and introduction of the additional charged H-bond donor enhances the H-bonding network (5.5 ± 1.7 bonds *vs.* 3.7 ± 1.4 for nisin_1–12_ averaged along the total MD time) in the nisin_1–12_(Dha5Lys)/DMPPi complex (Figure 4). The data suggest that the mutant may possess an improved lipid II binding properties *in vitro* and enhanced antibacterial effect *in vivo*.

## 4. Discussion

Based on this and previous work [27], we found a common pyrophosphate pharmacophore for type A lantibiotics recognition, characterized by directed into a single center H-bond donors, i.e., backbone NH groups of a cyclic peptide of optimal size. Here, we emphasize the importance of a positively charged group for PPi binding, which is placed outside of the ring structure and is introduced by either *N*-terminus or a side chain. Within the investigated type A lantibiotics, ring B plays an essential role in DMPPi binding, stabilizing conformation of ring A *via* one or two H-bonds rather than directly interacting with the target.

The key binding motif for PPi capture (co-oriented NH groups) was originally revealed in the NMR structure [21]. The recently defined X-ray structure of another lipid II’s PPi targeting AMP—depsipeptide teixobactin analogue bound to the chloride anion—also contains a very similar “cavity” fulfilled with amide protons [21,41].

This “key pharmacophore” can be adjusted, for example, by modification of backbone torsion angles and a side chain composition. In this work, we showed how both of these possibilities deliver the advantage for epidermin_1–12_ and gallidermin_1–12_ over nisin_1–12_, and demonstrated that, *in silico*, these features can be introduced into the nisin_1–12_ structure by a single point mutation (Dha5Lys), yielding the peptide with improved characteristics.

We expect that other lantibiotics and AMPs that specifically recognize pyrophosphate (mersacidin, teixobactin) should act in a similar way, yet inevitably contain individual nuances. Their establishment is the goal of future experiments and calculations.

## 5. Conclusions

Our current results are in a good agreement with earlier published data on nisin_1–11_ [27]. Previously, we demonstrated that the spatial structure and mode of binding of nisin to the membrane-embedded lipid II significantly depend on the environment. We identified the complex-forming conformation of nisin_1–11_ in aqueous solution (Figure 1B), which is identical to the above-described NB1 state. Here, we extend this hypothesis and expect this conformation to be a common pyrophosphate binding motif across the lantibiotics with a similar A/B ring system.

In addition, we discovered the molecular-level features that may explain the experimental data, suggesting higher binding affinity of gallidermin and epidermin as compared to nisin [26]: (1) nisin’s unsaturated Dha residue renders ring A more rigid and less capable of forming intermolecular h-bonds to PPi; (2) the presence of additional H-bond donors, i.e., side chain of Lys-4 residue in epidermin and gallidermin, contribute to PPi binding. These findings can be used to further study lipid II recognition by antimicrobial (poly)cyclic peptides and to develop new antibiotics.

## Figures and Tables

**Table 1:** MD simulations conducted in this work. Each line is a system composition with a number of entities given in parenthesis and subscript, and MD length with a number of independent replicas.

System Composition	MD Duration, ns
*Peptides_1–12_ in solution*	
Nisin_1–12 (1)_/Water_(5694)_/Cl^−^_(1)_	5 × 500
Epidermin_1–12 (1)_/Water_(5687)_/Cl^−^_(1)_	5 × 500
Gallidermin_1–12 (1)_/Water_(5687)_/Cl^−^_(1)_	5 × 500
*Peptides_1–12_ with DMPPi in solution*	
Nisin_1–12 (1)_/DMPPi_(1)_/Water_(6707)_/Na^+^_(1)_	5 × 500
Epidermin_1–12 (1)_/DMPPi_(1)_/Water_(6706)_/Na^+^_(1)_	5 × 500
Gallidermin_1–12 (1)_/DMPPi_(1)_/Water_(6707)_/Na^+^_(1)_	5 × 500
Nisin_1–12_(Dha5Lys)_(1)_/DMPPi_(1)_/Water _(5690)_	4 × 500
Nisin_1–12 (1)_/DMPPi_(3)_/Water_(5671)_/Na^+^_(5)_	5 × 500
Epidermin_1–12 (1)_/DMPPi_(3)_/Water_(5661)_/Na^+^_(5)_	5 × 500
Gallidermin_1–12 (1)_/DMPPi_(3)_/Water_(5670)_/Na^+^_(5)_	5 × 500
Nisin_1–12_(Dha5Lys)_(1)_/DMPPi_(3)_/Water_(5673)_/Na^+^_(4)_	4 × 500

## Supplementary Materials

The following are available online at www.mdpi.com/article/10.3390/mi12101169/s1, Figure S1: Structures of the characteristic for lantibiotics amino acids lanthionine (Lan), methyllanthionine (MeLan), dehydroalanine (Dha) and dehydrobutyrine (Dhb); Figure S2: Conformational analysis of the peptides in the unbound state; Figure S3: RMSF of rings A and B in unbound/bound states; Figure S4: Spontaneous formation of nisin_1–12_/DMPPi complexes in MD trajectories with 1 DMPPi; Figure S5: Spontaneous formation of epidermin_1–12_/DMPPi complexes in MD trajectories with 1 DMPPi; Figure S6: Spontaneous formation of gallidermin_1–12_/DMPPi complexes in MD trajectories with 1 DMPPi; Figure S7: Spontaneous formation of nisin_1–12_/DMPPi complexes in MD trajectories with 3 DMPPi; Figure S8: Spontaneous formation of epidermin_1–12_/DMPPi complexes in MD trajectories with 3 DMPPi; Figure S9: Spontaneous formation of gallidermin_1–12_/DMPPi complexes in MD trajectories with 3 DMPPi; Figure S10: Conformational analysis of the peptides in the DMPPi-bound states; Figure S11: Intramolecular hydrogen bonds heatmap in the DMPPi-bound states: NB1, EB1, and GB1.
