# Supplementary material for: Molecular Dynamics Insight into the Lipid II Recognition by Type A Lantibiotics: Nisin, Epidermin, and Gallidermin
Source: Micromachines (Basel). 2021 Sep 28;12(10):1169. doi: 10.3390/mi12101169 (PMC8538299; doi:10.3390/mi12101169)
Supplement: Supplementary file 1 [file micromachines-12-01169-s001.zip › micromachines-1383097-supplementary.pdf]

Supplementary information

Molecular dynamics insight into the lipid II recognition by type A lantibiotics: nisin, epidermin and gallidermin

I. Panina[1,2], A. Taldaev[3,4], R. Efremov[1,2,*], and A. Chugunov[1,2]

[1] Shemyakin-Ovchinnikov Institute of Bioorganic Chemistry, Russian Academy of Sciences, 16/10 Miklukho-Maklaya St., Moscow, 117997, Russia
[2] National Research University Higher School of Economics, Moscow, 101000, Russia
[3] Sechenov First Moscow State Medical University (Sechenov University), 8-2 Trubetskaya St., Moscow, 119991, Russia
[4] V.N. Orekhovich Institute of Biomedical Chemistry, 10-8 Pogodinskaya St., Moscow, 119121, Russia

*Corresponding author. Email: batch2k@yandex.ru

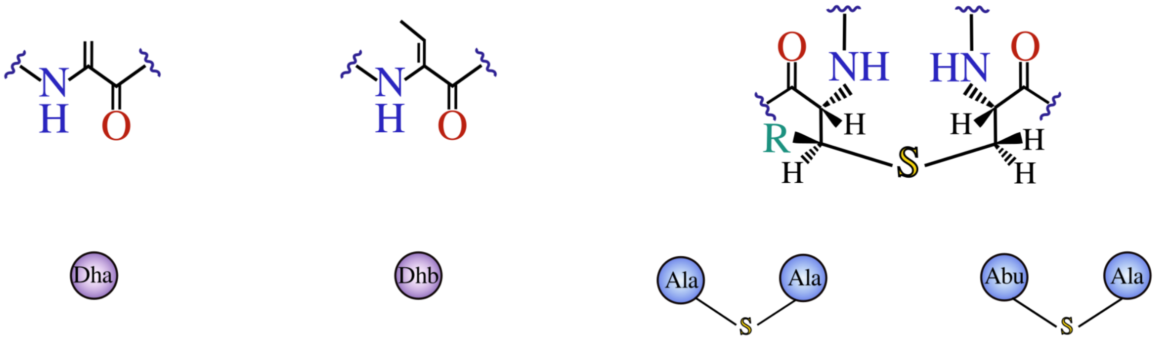

Dehydroalanine (Dha) | Dehydrobutyrine (Dhb) | Lanthionine (Lan) **R = H** | Methyllanthionine (MeLan) **R = CH_3**

Supplementary Figure S1. Structures of the characteristic for lantibiotics amino acids lanthionine (Lan), methyllanthionine (MeLan), dehydroalanine (Dha) and dehydrobutyrine (Dhb).

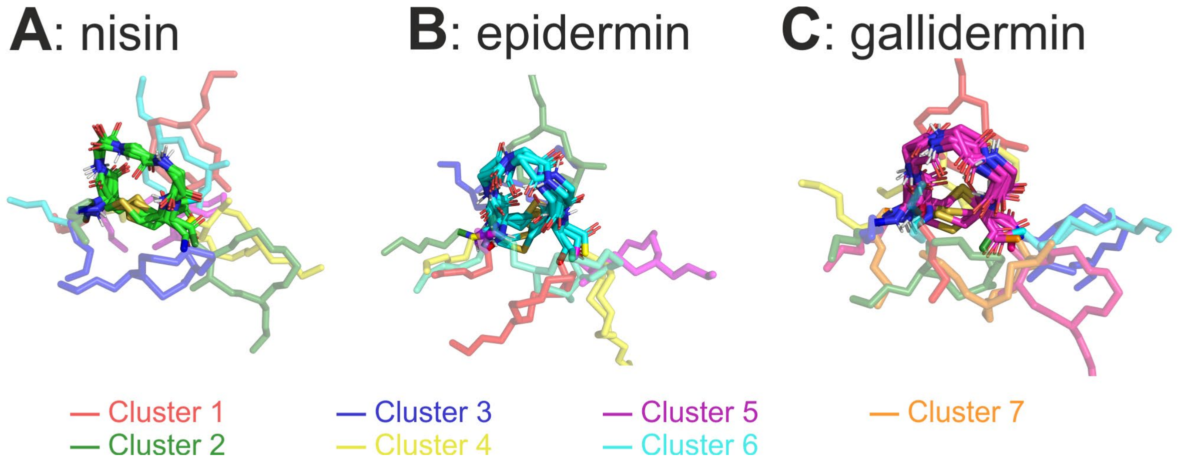

Supplementary Figure S2. Conformational analysis of the peptides in the unbound state. **(A)** $Nisin_{1–12}$ states N1–6 with the following population: 52.6%; 29.7%; 7.4%; 4.5%; 2.6%; 1.5%. **(B)** $Epidermin_{1–12}$ states E1–6: 39.8%; 34.9%; 11.7%; 4.7%; 4.1%; 3.1%) . **(C)** $Gallidermin_{1–12}$ states G1–7: 34.5%; 26.7%; 16%; 10.2%; 4.1%; 3.0%; 1.5%. The structures are superimposed by the ring A backbone (carbon atoms are shown with *green sticks* for nisin, *cyan* for epidermin and *magenta* for epidermin).

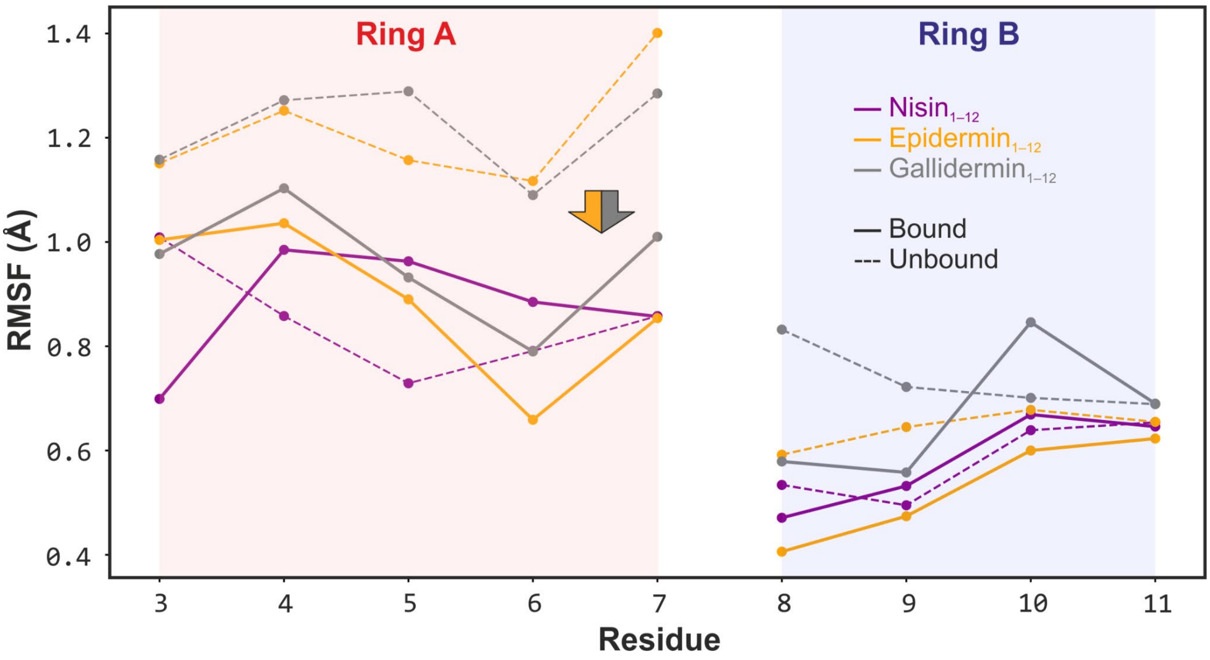

Supplementary Figure S3. RMSF of peptides' rings A and B in unbound/bound states along MD trajectories (nisin$_{1-12}$ (*purple*), epidermin$_{1-12}$ (*orange*) and gallidermin$_{1-12}$ (*grey*) in presence (*solid lines*) and absence (*dashed lines*) of DMPPi). The notable reduction in flexibility of epidermin$_{1-12}$ and gallidermin$_{1-12}$ ring A upon DMPPi binding is highlighted with an arrow.

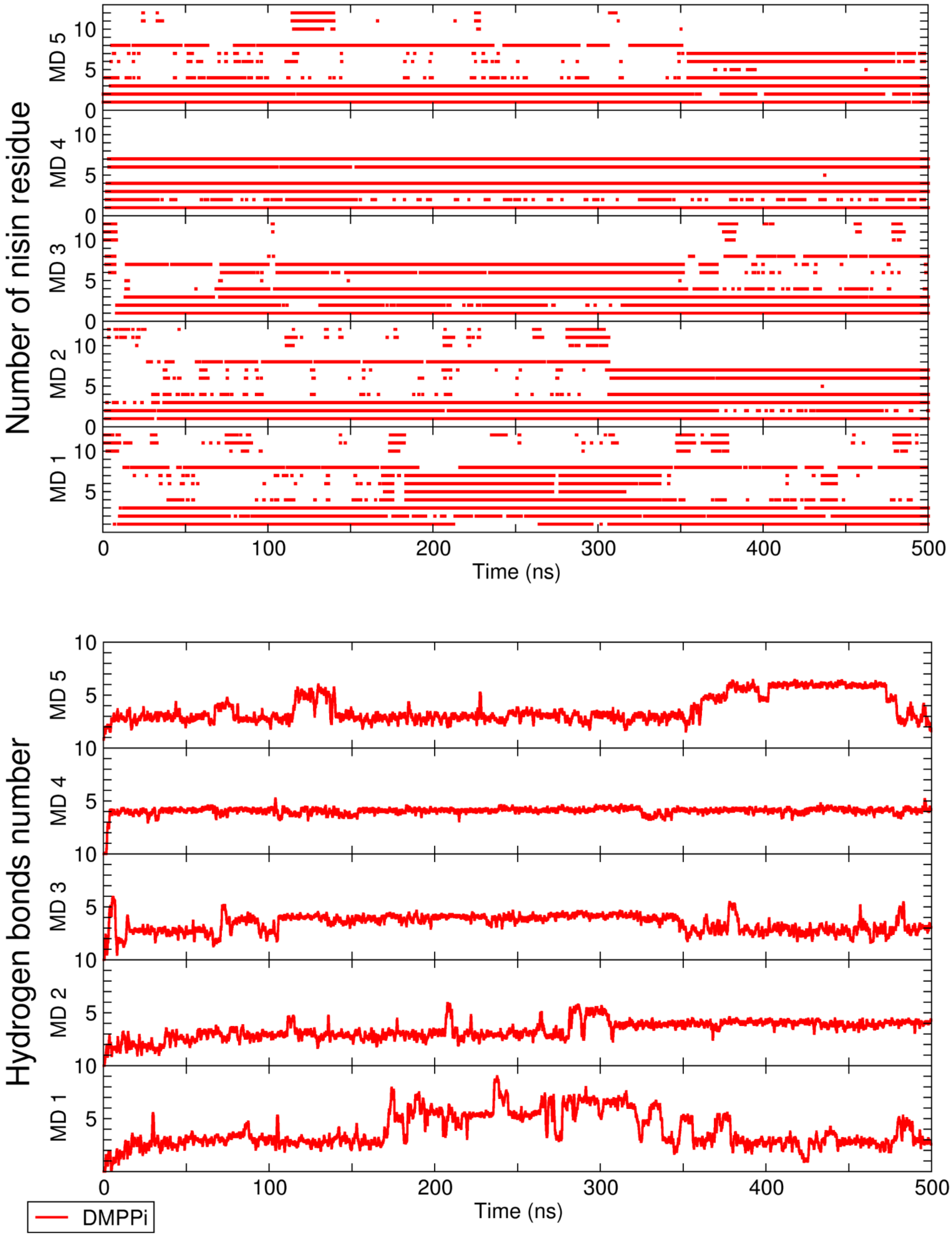

Supplementary Figure S4. **Spontaneous formation of nisin$_{1-12}$/DMPPi complexes in MD trajectories with 1 DMPPi.** ***Upper panel:*** Hydrogen bonds maps between peptide backbone and DMPPi ion. Each dot indicates H-bond between DMPPi and particular nisin$_{1-12}$ residue (*vertical axis*) at a given MD time (*horizontal axis*). ***Lower panel:*** Time-averaged (0.1 ns window) number of simultaneous H-bonds between nisin$_{1-12}$ and DMPPi. Five independent MD replicas are shown as five vertically stacked plots.

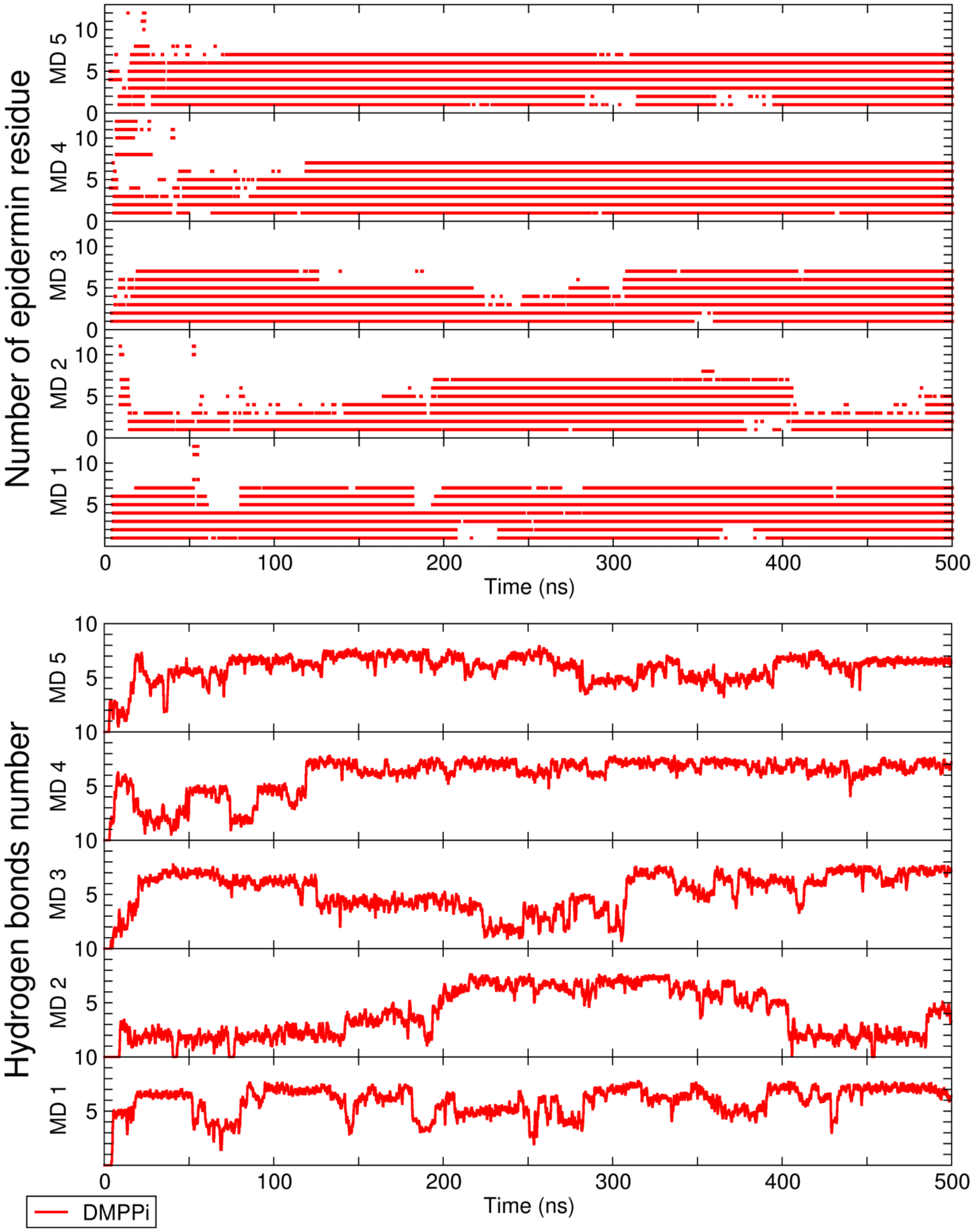

Supplementary Figure S5. Spontaneous formation of epidermin$_{1-12}$/DMPPi complexes in MD trajectories with 1 DMPPi. ***Upper panel:*** Hydrogen bonds maps between the peptide backbone and DMPPi ion. Each dot indicates H-bond between DMPPi and particular epidermin$_{1-12}$ residue (*vertical axis*) at a given MD time (*horizontal axis*). Note the continuous involvement of the 5th residue in PPi binding. ***Lower panel:*** The time-averaged (0.1 ns window) number of simultaneous H-bonds between epidermin$_{1-12}$ and DMPPi. Five independent MD replicas are shown as five vertically stacked plots.

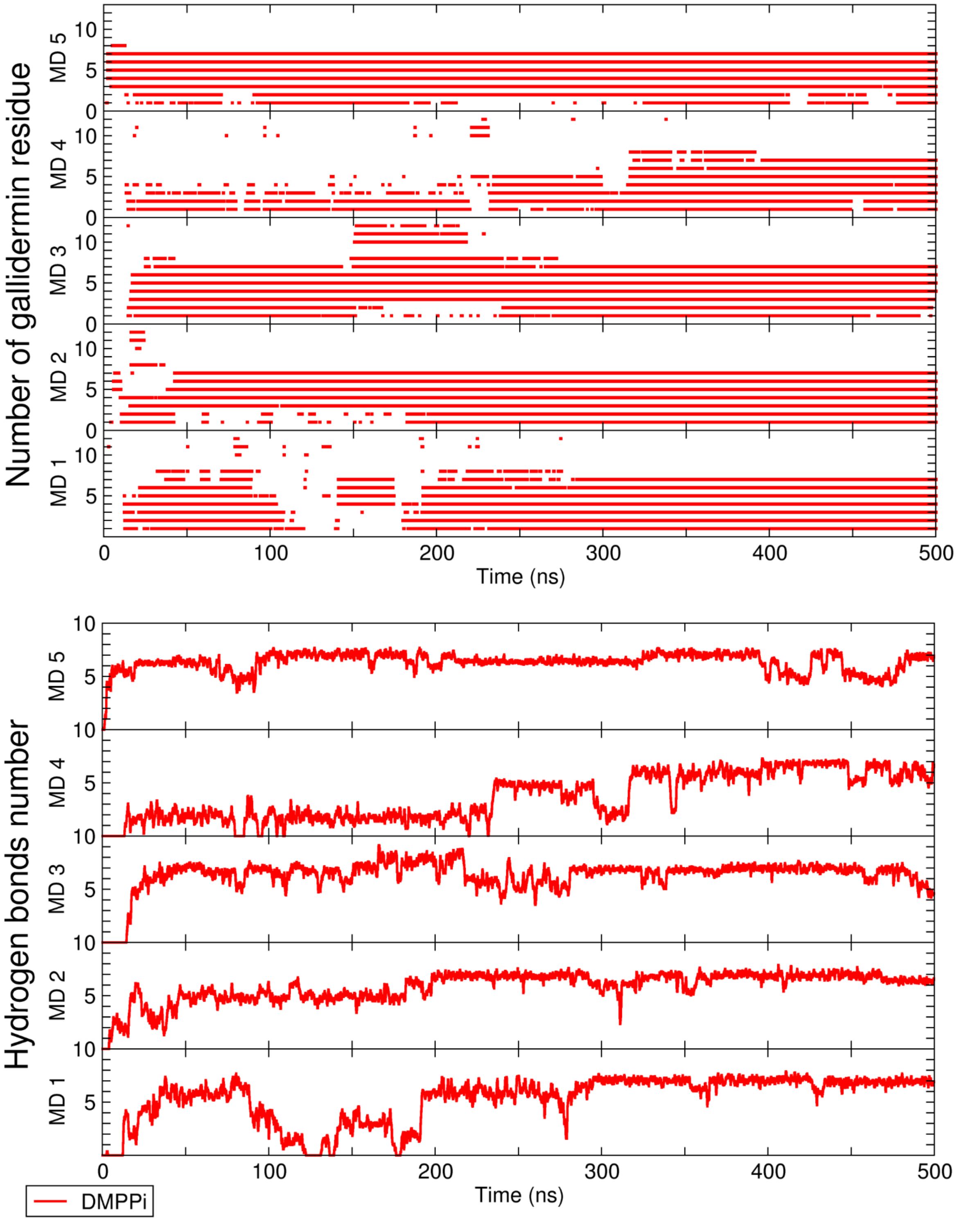

Supplementary Figure S6. **Spontaneous formation of gallidermin$_{1\text{–}12}$/DMPPi complexes in MD trajectories with 1 DMPPi.** ***Upper panel:*** Hydrogen bonds maps between peptide backbone and DMPPi. Each dot indicates H-bond between DMPPi and particular gallidermin$_{1\text{–}12}$ residue (*vertical axis*) at a given MD time (horizontal axis). Note the continuous involvement of the 5th residue in PPi binding. ***Lower panel:*** The time-averaged (0.1 ns window) number of simultaneous H-bonds between gallidermin$_{1\text{–}12}$ and DMPPi. Five independent MD replicas are shown as five vertically stacked plots.

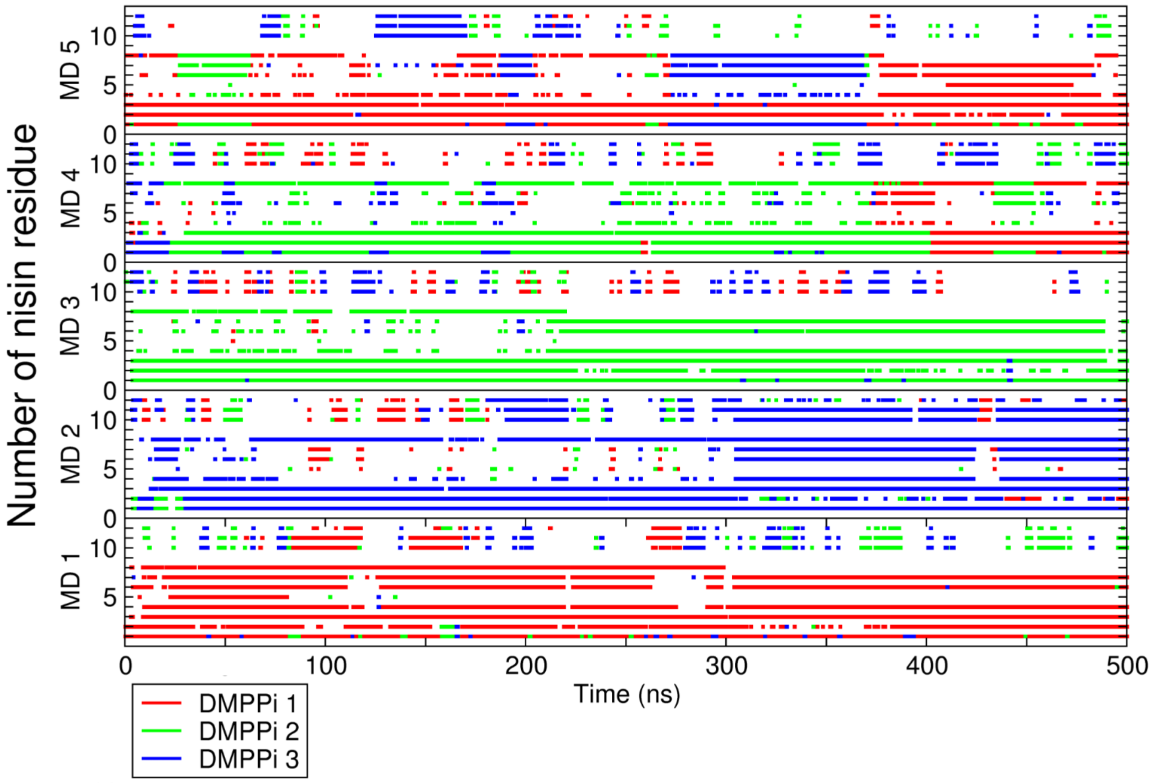

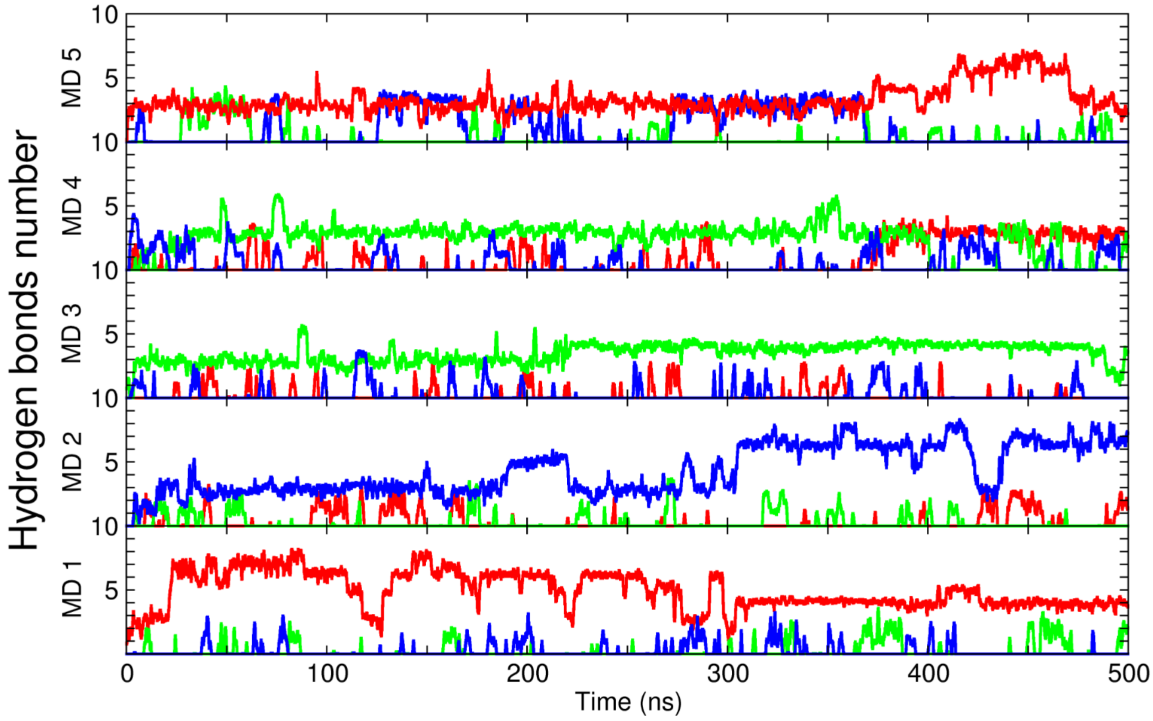

Supplementary Figure S7. **Spontaneous formation of nisin$_{1–12}$/DMPPi complexes in MD trajectories with 3 DMPPi.** ***Upper panel:*** Hydrogen bonds maps between peptide backbone and three DMPPi ions. Each dot indicates H-bond between different DMPPi ions (*different colors*) and particular nisin$_{1–12}$ residue (*vertical axis*) at a given MD time (*horizontal axis*). For most of MD time, one DMPPi ion is trapped by nisin$_{1–12}$. Note the rare and unstable events of the second PPi capturing by ring B (residues 8–11). ***Lower panel:*** Time-averaged (0.1 ns window) number of simultaneous H-bonds between nisin$_{1–12}$ and DMPPi. Five independent MD replicas (see Table 1) are shown as five vertically stacked plots.

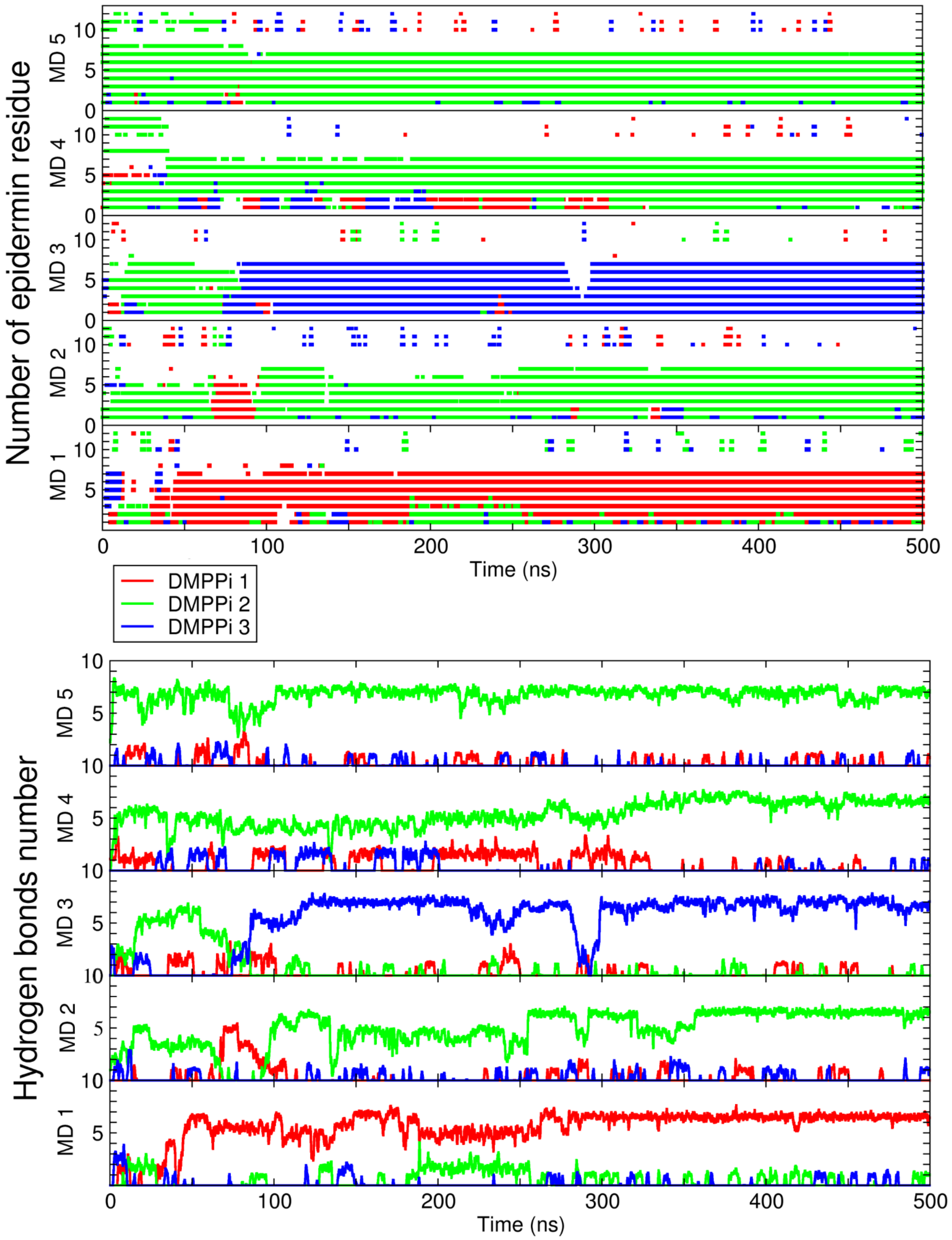

Supplementary Figure S8. Spontaneous formation of epidermin$_{1-12}$/DMPPi complexes in MD trajectories with 3 DMPPi. ***Upper panel:*** Hydrogen bonds maps between the peptide backbone and three DMPPi ions. Each dot indicates H-bond between different DMPPi ions (*different colors*) and particular epidermin$_{1-12}$ residue (*vertical axis*) at a given MD time (*horizontal axis*). Note the continuous involvement of the 5th residue in PPi binding. ***Lower panel:*** The time-averaged (0.1 ns window) number of simultaneous H-bonds between epidermin$_{1-12}$ and DMPPi. Five independent MD replicas are shown as five vertically stacked plots.

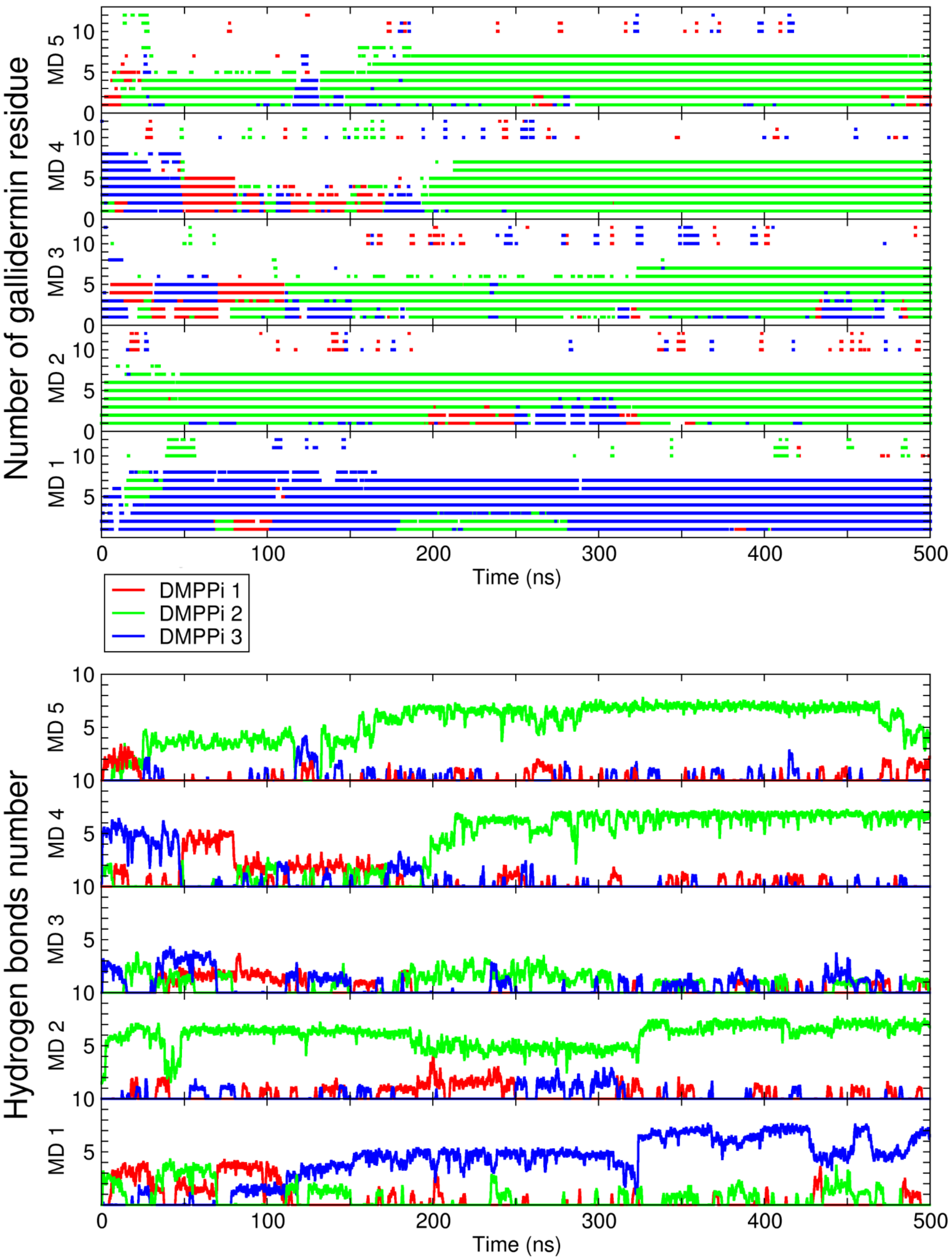

Supplementary Figure S9. **Spontaneous formation of gallidermin$_{1\text{–}12}$/DMPPi complexes in MD trajectories with 3 DMPPi.** ***Upper panel:*** Hydrogen bonds maps between peptide backbone and three DMPPi ions. Each dot indicates H-bond between different DMPPi ions (*different colors*) and particular gallidermin$_{1\text{–}12}$ residue (*vertical axis*) at a given MD time (horizontal axis). Note the continuous involvement of the 5th residue in PPi binding. ***Lower panel:*** The time-averaged (0.1 ns window) number of simultaneous H-bonds between gallidermin$_{1\text{–}12}$ and DMPPi (different colors). Five independent MD replicas are shown as five vertically stacked plots.

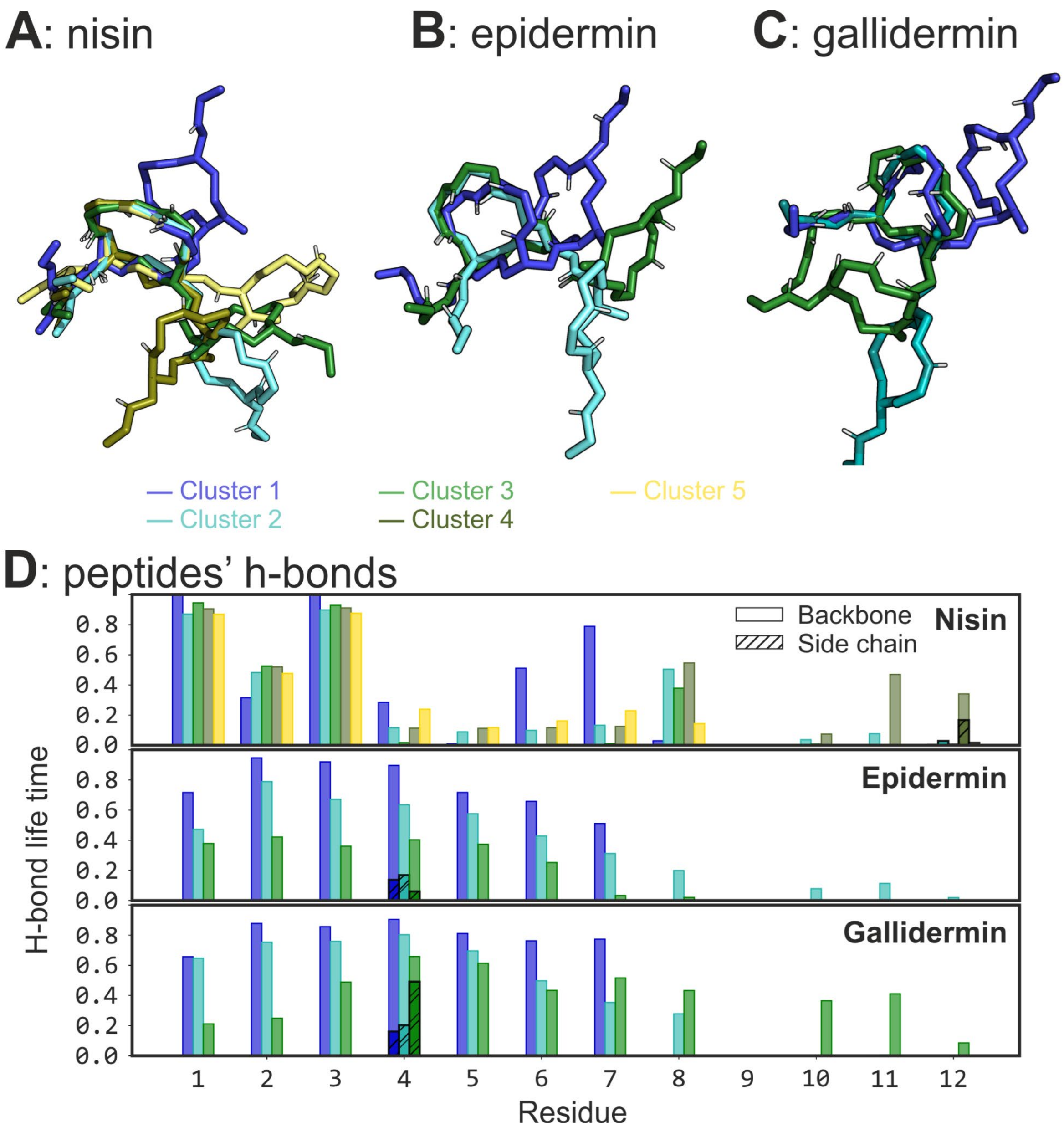

Supplementary Figure S10. Conformational analysis of the peptides in the DMPPi-bound states. (A) Nisin$_{1-12}$ forms five major states (population: 51.1%; 36.2%; 4.2%; 3.1%; 2%). **(B)** Epidermin$_{1-12}$ exists in a total of three conformations (89.2 %; 3.7 %; 1.9 %). **(C)** Gallidermin$_{1-12}$ adopts three preferred states (73%; 12.3%; 2%). The structures are superimposed by the ring A backbone. Note the reduced conformational space of each peptide as compared to unbound ensemble (Fig. S2). **(D)** Intermolecular H-bonds revealed in major peptides conformations. Relative H-bonds frequencies formed by amino acids backbone are solid-colored and by the side chain with hatching. Modes are numbered as cluster population decreases. The provided conformations snapshots and clusters percentages correspond to trajectories with 1 DMPPi.

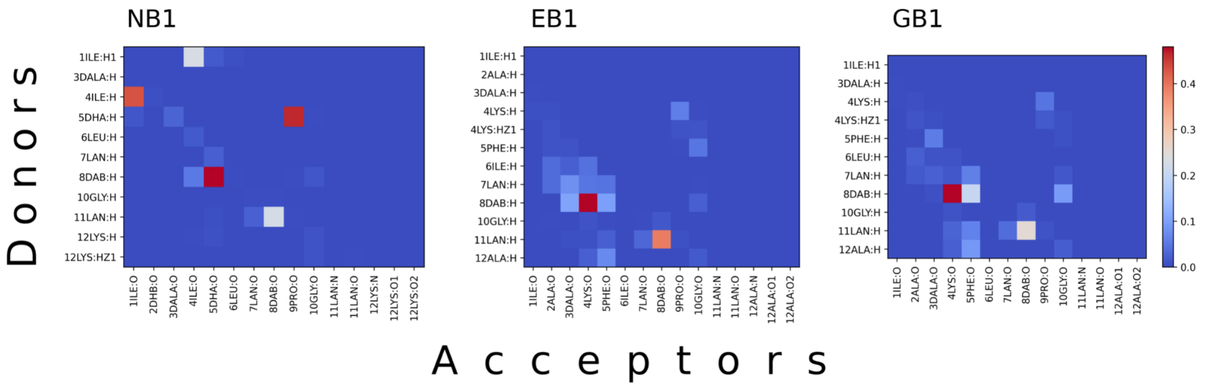

Supplementary Figure S11. **Intramolecular hydrogen bonds heatmap in the DMPPi-bound states: NB1, EB1, and GB1.** The titles of the atoms correspond to the generally accepted name in RCSB Protein Data Bank format. Only hydrogen bonds acceptor and donor atoms are shown. The colorbar indicates hydrogen bond lifetime (as a fraction of MD time).
